# Intervention is a better predictor of tDCS mind-wandering effects than subjective beliefs about experimental results

**DOI:** 10.1038/s41598-022-16545-0

**Published:** 2022-07-30

**Authors:** Matilda S. Gordon, Jennifer X. W. Seeto, Paul E. Dux, Hannah L. Filmer

**Affiliations:** School of Psychology, The University of Queensland, McElwain Building (24A), St Lucia, QLD 4072 Australia

**Keywords:** Psychology, Cognitive neuroscience, Attention

## Abstract

Blinding in non-invasive brain stimulation research is a topic of intense debate, especially regarding the efficacy of sham-controlled methods for transcranial direct current stimulation (tDCS). A common approach to assess blinding success is the inclusion of correct guess rate. However, this method cannot provide insight into the effect of unblinding on observed stimulation outcomes. Thus, the implementation of measures to systematically evaluate subjective expectation regarding stimulation is needed. Previous work evaluated subjective effects in an earlier study which reported a mind-wandering and tDCS data set and concluded that subjective belief drove the pattern of results observed. Here we consider the subjective and objective intervention effects in a key contrast from that data set—2 mA vs. sham—which was not examined in the reanalysis. In addition, we examine another key contrast from a different tDCS mind-wandering study that employed similar methodology. Our findings support objective intervention as the strongest predictor of the observed effects of mind-wandering in both re-analyses, over and above that of subjective intervention. However, it is important to control for and understand the possible inadequacies of sham-controlled methods.

## Introduction

Non-invasive brain stimulation (NIBS) is a popular tool for investigating causal relationships between activity in cortical regions and behaviour^1^. In 2020 the brain stimulation industry was projected to be worth an estimated three billion dollars^2^, a figure that is almost certain to increase in the future. There are multiple methods of NIBS, one of the most common being transcranial direct current stimulation (tDCS^3^). tDCS typically involves passing electrical current through two electrodes placed on the scalp; a cathode and an anode^4^. Interest has grown in this brain stimulation approach as it can lead to cognitive enhancement both within clinical settings, such as for the treatment of drug-resistant depression^5,6^, as well as in commercial settings via do-it-yourself devices such as the *foc.us* headset^7–9^. A variety of cognitive operations have previously been shown to be influenced by tDCS including: motor^10^ and speech motor learning^11^, working memory^12,13^, response selection^14^, multitasking^15^, and attention^16,17^. Given this broad interest there is growing demand for clarity on the efficacy of brain stimulation across the extensive stimulation parameter space.

Although tDCS has been employed extensively, some reviews and meta-analyses^18–20^ have suggested limited to no effects of transcranial stimulation, with a key focus being on large variability across participants. For example, Lopez-Alonso et al.^19^ found only 45% of participants (n = 56) responded to anodal transcranial current stimulation as expected. In an opinion piece, Filmer et al.^3^ provide recommendations to insure the reliability, reproducibility, and validity of effects of NIBS studies. Key factors identified by the authors to be addressed include poor methodological design, under-powered samples which give rise to inflated results and, most importantly for the present study, the inadequate blinding of control conditions.

The most common method of blinding in tDCS studies is the sham control method^21^, which mimics the typical initial sensations (i.e., itching or tingling) induced by tDCS by delivering active stimulation for a short period at the beginning (and sometimes again at the end), then either no stimulation or very reduced pulses (which allow some continued sensation) for the rest of the session. Typically, the length of this period of active stimulation is dependent on the stimulation length used for the active condition^22^. It is assumed that sham stimulation controls for any potential unrelated effects of the direct cortical stimulation^22^. However, the efficacy of sham-controlled approaches has been called in to question^23–25^. Indeed, in NIBS studies, participants can report perceptual sensations such as visual disturbances, and cutaneous feelings^26,27^. Cutaneous feelings are common in tDCS as seen in a prospective comparison conducted by Kessler et al.^26^ with 131 subjects in 277 tDCS sessions. Such feelings were significantly higher in active conditions as compared to sham, for example tingling (89% active vs. 53% sham) and itching (81% vs. 42%). This is potentially a major issue for sham-controlled studies, as observed results may reflect peripheral effects rather than the influence of stimulation on the cortex. Or, put differently, failure of the blinding condition (although, see^28^ and^29^). Another factor that may add to blinding inefficiency is the inadequate reporting of adverse events (such as cutaneous feelings), as this is not only a safety concern but also prevents the experimenter from gaining an understanding of the strength of blinding^27^. Indeed, Wallace et al.^30^ found that when investigating the comfort and efficacy of sham-blinding, after a second session of tDCS participants were able to correctly guess stimulation above chance (65%).

A recent paper published by Fassi and Kadosh^31^ highlighted possible inadequacies in a common blinding practice in sham-controlled studies, referencing an example from Schecklmann et al.^32^, whilst also bringing attention to studies by Blumberger et al.^33^ and Filmer et al.^4^ to demonstrate how correct guess rate—the percentage of participants who correctly guess their experimental group—is not an accurate measure of blinding success. Specifically, a hypothetical NIBS experiment was posited, whereby the correct guess rate for both active and sham is 75%, meaning a large majority of the sham condition would have correctly recognised that they did not receive active stimulation^31^, thus demonstrating unblinding of participants. The authors highlight active stimulation guess rate—the percentage of participants who correctly guess they have received active treatment—as a more effective measure of indicating successful blinding, or its failure^31^.

However, a key limitation of active stimulation guess rate (or any guess rate measure) is the inability to reveal the effect blinding (or unblinding) has on the effects and results seen in studies featuring blinding controls, nor does it reveal the direction of these effects. A participant in a sham condition, who believes they are privy to their stimulation condition, could either try to enhance their performance, limit their performance, or have no change in behaviour^34^. A measure of guess rate cannot determine whether this has occurred as they can only reveal the *possibility* of behavioural changes due to unblinding. Follow-up analyses are needed to investigate these possibilities.

Such a systematic evaluation of subjective expectation of stimulation can be used to address the impact of beliefs regarding stimulation on performance^34^. For example, Fassi and Kadosh^35^ used a method of evaluating the effects of participant subjective belief regarding stimulation conditions (i.e. active or sham condition and stimulation intensity), via open-source data from a tDCS mind-wandering study we conducted^4^. The present study also employs this data for a re-analysis, as well as a second mind-wandering study given its methodological similarities^36^.

The two mind-wandering studies featuring tDCS from our lab group^4,36^ implemented a sustained attention to response task (SART) with thought probes scattered throughout trials to investigate mind-wandering. Filmer et al.^4^ investigated the effect of polarity (anodal and cathodal) and intensity (1 mA and 2 mA) on mind-wandering via tDCS to the left prefrontal cortex, whilst Filmer et al.^36^ used the same methodology, polarities, and intensities to investigate the influence of active stimulation to both the prefrontal cortex (PFC) and the inferior parietal lobule (IPL) simultaneously. Both studies measured participant experience after each stimulation session with two questions; whether participants believe they received active or sham stimulation (as a binary choice), and whether they believed they received sham (not applicable), weak, moderate, or strong stimulation.

The key findings for both studies demonstrated modulation of mind-wandering after stimulation; specifically, in Filmer et al.^4^ moderate evidence for increased average task-unrelated thoughts (TUT-ratings) after 2 mA cathodal stimulation relative to sham^4^. The two other active conditions cathodal 1 mA and anodal 1 mA failed to produce meaningful results of stimulation on average TUT-ratings, and cathodal 1.5 mA provided anecdotal evidence. For the study by Filmer et al.^36^, anodal stimulation to the left PFC and cathodal stimulation to the right IPL simultaneously at 1 mA produced moderate increases in TUT-ratings relative to sham^36^. Interestingly in this second study, cathodal stimulation to the left PFC and anodal stimulation to the right IPL at 2 mA produced at best anecdotal evidence of an effect of stimulation as compared to sham.

Fassi and Kadosh^35^ re-analysed Filmer et al.^4^ including the subjective information relating to blinding in the analyses. Specifically, Bayesian ANOVAs compared objective and subjective interventions for mind-wandering across *all* conditions. They concluded that subjective intervention was a better predictor of participant performance than objective intervention, or a combination of the two^35^. Particularly, those who believed they had received active stimulation had higher levels of mind wandering than those who answered sham. A similar pattern was observed when investigating tDCS dosage: as subjective dosage increased, so did average mind-wandering score in a proportional manner. Thus, this finding seemingly calls into question the conclusions of Filmer et al.^4^ and, more broadly, those of any study with purely a sham control. However, a key limitation of the Fassi and Kadosh^35^ approach was that they examined all conditions on the Filmer et al.^4^ study in a single analysis, whereas only effects were observed for cathodal 2.0 mA stimulation as compared to sham comparison with moderate evidence (see Table 1 for specific BF_10_ values for the experimental conditions from Filmer et al.^4^).

**Table 1:** Individual BF_10_ and percent error for each stimulation condition (as compared to sham) for average task-unrelated thought across all experimental trials for Filmer et al.^4^.

Stimulation condition	BF_10_	Error %
Anodal 1.0 mA	0.781	0.010
Cathodal 1.0 mA	0.540	0.007
Cathodal 1.5 mA	2.189	0.005
Cathodal 2.0 mA	7.436	8.694e−5

The present study sought to re-analyse two existing data sets on mind wandering^4,36^, to account for both subjective and objective beliefs. Here, we used a similar approach as Fassi and Kadosh^35^ but with more directed t-tests/paired comparisons, as had been conducted in the two original papers^4,36^. To preview the results, for both re-analyses the objective information regarding stimulation type (active or sham) and dosage (stimulation intensity) was shown to be the best predictor of the influence of tDCS on mind wandering above subjective, or a combination of objective and subjective intervention.

## Reanalysis 1: Filmer et al.^4^

### Method

#### Data and materials

The study by Filmer et al.^4^ was pre-registered via the Open Science Framework with details of the analysis plan, methodology and sample size (https://osf.io/j6mqa/). The raw data from the original study has been made open source and can be accessed via the UQ eSpace^37^. Experimental materials, such as the task, can also be accessed here as well as the demographic and questionnaire data used to establish the experimental measures. For a full overview of the original methods, refer to Filmer et al.^4^.

#### Subjects

One hundred and fifty subjects (mean age = 23, SD = 5, 96 females) participated in the study. All subjects were right-handed with normal or corrected to normal vision. Subjects were assigned to one of five different stimulation groups based on their participant number sequentially (subject 1, 6, 11 etc. were assigned to anodal stimulation). The final sample per group was 30^4^.

#### Task

Subjects completed a sustained attention-to-response task (SART), responding via a keyboard key press (space bar) to non-target stimuli (any number except 3). In each trial, a stimulus was presented in the centre of the display. Subjects were to withhold their answer when the target stimulus was presented (the number 3). Stimuli were presented for 1 s and a 1.2 s blank screen appeared between stimuli. The background of the display was light grey (RGB: 104, 104, 104), the stimuli were black (RGB: 255, 255, 255), in size 40 font. Trials consisted of an average of 20 non-target stimuli (*SD* = 5.69). At the end of half of the trials a target stimulus was presented and in the other half an unrelated thought probe was presented in the centre of the display in size 20 black font (1° visual angle). Task un-related thought (TUT) probes asked: “*To what extend have you experienced task unrelated thoughts prior to the thought probe? 1 (Minimal)–4 (Maximal)”*. The corresponding numbers on a keyboard were used to indicate their response. Subjects undertook two practice trials prior to stimulation (one target and one thought probe). A total of 48 trials were completed after stimulation with 24 target and 24 thought probes, split into 8 blocks (6 trials each) with approximately three of each trial type randomly intermixed.

#### tDCS

tDCS was administered using a Neuroconn stimulator via two 5 × 5 cm saline-soaked electrodes. The reference electrode was located on the right orbito-frontal region and the target electrode was placed over F3 (EEG 10–20 system). The four active stimulation groups consisted of various polarity and intensity combinations: anodal 1.0 mA, cathodal 1.0 mA, cathodal 1.5 mA, and cathodal 2.0 mA. In these conditions, total stimulation duration was 20 min including a 30 s ramping up and down period. Those in the sham stimulation condition also received a 30 s ramping period, but only received 15 s of active stimulation before stimulation ramped down for a further 30 s (thus a total of 1 min and 15 s of stimulation). During stimulation, subjects were asked to sit quietly with their eyes open.

#### Measures

##### Objective intervention

Objective intervention features sham, cathodal 1.0 mA, anodal 1.0 mA, cathodal 1.5 mA and cathodal 2.0 mA conditions, whilst objective dosage features sham (or N/A), weak, moderate, and strong. The conditions for each variable overlap i.e., those that received sham stimulation also fall into the sham (N/A) dosage condition, those in anodal 1.0 mA and cathodal 1.0 mA in the weak dosage condition, cathodal 1.5 mA in the moderate condition and finally cathodal 2.0 mA in the strong condition. Thus, there are four conditions for objective dosage and five for objective intervention; however, it should be noted that in this configuration two objective intervention conditions fall into the ‘weak’ dosage condition. This would result in the grouping of anodal and cathodal stimulation protocols. As previous tDCS studies have demonstrated differential effects of polarity (see^38^ and^39^), the combination of these conditions into the ‘weak’ category would pose a potentially significant problem given the possibility of these conditions having differential effects on mind-wandering thus, we chose to separate them. Given these considerations, objective intervention and objective dosage have the same participants in the same categories, thus can be used interchangeably.

##### Subjective intervention

Subjective intervention refers to the participants subjective belief regarding the stimulation condition they have been assigned to; either sham or active.

##### Subjective dosage/intensity

Subjective dosage refers to the perceived strength of stimulation participants believe they have received. Participants indicated that they believed they either received sham, weak, moderate, or strong stimulation.

#### Statistical analysis

As with both Filmer et al.^4^ and Fassi and Kadosh^35^ all statistical analysis was conducted in JASP (version 0.14.1 for MacOS^40^). Analyses were conducted on the open-access dataset from Filmer et al.^41^ using average mind wandering scores calculated from the whole experimental session for each subject.

To verify both the data and the coding were reproducible, we implemented the statistical analyses of both Fassi and Kadosh^35^ and Filmer et al.^4^. All previous findings were replicated. Bayesian statistics and their frequentist counterparts were used as both were included in the previous papers. BF_10_ values above 1 provide evidence of H_1_ over H_0_; values below BF_10_ 1 (or BF_01_) suggest evidence of H_0_ over H_1_. Conventionally, the strength of evidence for a particular hypothesis compared to the competing hypothesis is only regarded as noteworthy when BF_10_ values are above 3 and thus any results that fall between 1 and 3 are inconclusive^42^. Often, this is referred to as ‘anecdotal evidence’ for any given hypothesis, and thus throughout this paper these values will be referred to as such. Values of BF_10_ ~ 1 will be considered to provide no evidential value for H_1_ over H_0_. BF_01_ values 1–3 will be considered as ‘anecdotal evidence’ for the null hypothesis over H_1_, BF_01_ 3–10 values as moderate evidence and lastly BF_01_ > 10 as strong evidence. When considering values in favour of H_1_ over H_0_, as outlined above BF_10_ values falling between 1 and 3 will be considered inconclusive or anecdotal (and thus should be interpreted with caution), BF_10_ values 3–10 as moderate evidence and BF_10_ values > 10 as strong evidence in favour of H_1_. As frequentist statistics were also evaluated, all values of *p* < 0.05 were accepted as statistically significant.

In JASP (using default priors) we replicated the Bayesian ANOVAs conducted by Fassi and Kadosh^35^ however, as previously outlined, only data from the cathodal 2.0 mA and sham condition were included in our key analysis. All relevant post-hoc pairwise comparisons were conducted following each analysis. The first Bayesian ANOVA included objective intervention and subjective intervention as between-subjects factors. To rule out the possibility of subjective information influencing performance in the absence of an effect, this analysis was also performed for each individual condition with no significant effect compared to sham (anodal 1.0 mA, cathodal 1.0 mA and cathodal 1.5 mA). The second Bayesian ANOVA employed objective intervention and subjective dosage as the between-subject factors. Although the authors conducted a third Bayesian ANOVA to investigate the effect of subjective dosage only, we did not include this given the results of the second ANOVA indicating subjective dosage has no evidential value of an effect on mind-wandering.

### Results

#### Summary of the results from Fassi and Cohen Kadosh

We replicated the analyses and findings of Fassi and Kadosh^35^. When comparing the effect of stimulation intervention, the key finding included subjective intervention (BF_inc_ = 2.442, BF_10_ = 3.374, *t*(148) = 2.55, *p* = 0.012) being the best predictor of the observed changes in mind-wandering over and above that of objective intervention as compared to sham. In short, following it appears based on this results that subjective participant belief regarding the type of stimulation received (sham versus active) provides a better explanation of participant performance compared to objective intervention, subjective and objective intervention combined or an interaction between the two. The full model result from this ANOVA can be found in the table located in Appendix A1.

For dosage, again, participant belief about stimulation dosage was a better predictor of mind wandering than objective intervention alone, a combination of the two measures or an interaction between them (BF_10_ = 3.708^35^). Fassi and Kadosh^35^ did not report the frequentist statistics for this finding however when we replicated the ANOVA we determined the values to be BF_inc_ = 2.685, BF_10_ = 3.713, *F*(3, 146) = 3.829, *p* = 0.011. The full model table can be found in Appendix A2. When investigating dosage alone, subjective dosage (BF_10_ = 5.911, *F*(3,198) = 4.198, *p* = 0.007) was also a better predictor of mind-wandering changes over and above that of objective intervention. We could not replicate the finding of BF_10_ = 5.911, instead finding BF_10_ = 3.713 and attribute this to a difference in data handling, given all other values were able to be replicated.

#### Effect of subjective belief on mind-wandering via 2.0 mA cathodal stimulation vs sham comparison

We implemented the first Bayesian ANOVA discussed above (employing objective intervention and subjective intervention as between-subject factors and average TUT as the outcome measure), however, crucially, we focussed exclusively on the cathodal 2.0 mA vs the sham comparison as this is the finding Filmer et al.^4^ focussed their conclusions on. The full model comparing against the null can be found in Appendix B1 however a summarised model table comparing these results to those of Fassi and Kadosh^35^ can be found in Table 2. Here, we observed that objective intervention was the strongest model predictor with moderate evidence (BF_inc_ = 7.437, BF_10_ = 7.436, *F*(1, 58) = 8.263, *p* = 0.006). Subjective intervention proved to be the least predictive within this model (BF_inc_ = 0.276, BF_10_ = 0.276, *F*(1, 58) = 0.104, *p* = 0.748). Post-hoc pairwise comparisons on the objective information indicated that cathodal 2.0 mA had increased mind-wandering (*M* = 2.288*,* BF_10_ = 7.436, *t*(59) = − 2.866, *p* = 0.006) as compared to sham (*M* = 1.772).

**Table 2:** Comparison of the summarised Bayesian ANOVA of objective intervention and subjective intervention findings from Fassi and Kadosh^35^ and the present study evaluating cathodal 2.0 mA stimulation only.

Models	BF_10_	All stimulation groups (Fassi and Kadosh^35^)		BF_10_	Cathodal 2.0 mA and sham	
		BF_inc_	Error %		BF_inc_	Error %
Null model	1.000			1.000		
Objective intervention	0.984	0.551	1.40 e^−4^	7.436	7.437	8.694 e^−5^
Subjective intervention	3.374	2.420	8.94 e^−8^	0.276	0.276	0.004
Objective + subjective intervention	1.492	0.197	2.201	2.133	0.365	2.627

When comparing objective intervention to subjective dosage, again objective intervention was the strongest predictor of mind-wandering within the model (BF_inc_ = 6.874, BF_10_ = 7.436, *F*(1, 58) = 8.263, *p* = 0.006). Subjective dosage was the weakest predictor in the model (BF_inc_ = 0.170, BF_10_ = 0.254, *F*(3, 56) = 0.847, *p* = 0.474). A comparison of these model findings compared to those found by Fassi and Kadosh^35^ can be found in Table 3, which demonstrates that when evaluating only conditions previously found to demonstrate meaningful results, the effect of subjective dosage can no longer be observed.

**Table 3:** Comparison of the summarised Bayesian ANOVA of objective intervention and subjective dosage findings from Fassi and Kadosh^35^ and the present study evaluating cathodal 2.0 mA stimulation only.

Models	BF_10_	All stimulation groups (Fassi and Kadosh^35^)		BF_10_	Cathodal 2.0 mA and sham	
		BF_inc_	Error %		BF_inc_	Error %
Null model	1.000			1.000		
Objective intervention	0.984	0.553	1.402 e^−4^	7.436	6.874	8.694 e^−5^
Subjective dosage	3.308	2.690	1.715 e^−4^	0.254	0.170	0.033
Objective + subjective dosage	1.658	0.161	0.587	1.203	0.510	2.011

#### Comparing non-significant conditions to sham

After analysing each individual non-significant condition against sham in separate Bayesian ANOVAs comparing objective and subjective intervention, the results indicate that in the absence of an objective effect, objective intervention remained the ‘best’ model predictor for anodal 1.0 mA (BF_inc_ = 0.706, BF_10_ = 0.781, *F*(1, 58) = 1.363, *p* = 0.248) and cathodal 1.5 mA (BF_inc_ = 1.701, BF_10_ = 2.189, *F*(1, 58) = 2.754, *p* = 0.103) compared to that of subjective intervention for the anodal 1.0 mA (BF_inc_ = 0.397, BF_10_ = 0.458, *F*(1, 58) = 0.105, *p* = 0.572) or cathodal 1.5 mA conditions (BF_inc_ = 0.514, BF_10_ = 0.788, *F*(1, 58) = 0.664, *p* = 0.419), although it should be noted that these BF values were anecdotal at best. Only within the cathodal 1.0 mA condition did subjective intervention (BF_inc_ = 0.672, BF_10_ = 0.735, *F*(1, 58) = 1.310, *p* = 0.257) provide a higher BF value than objective intervention (BF_inc_ = 0.548, BF_10_ = 0.540, *F*(1, 58) = 1.919, *p* = 0.171). However, given these BF values fall below 1 the null model is considered the best model predictor and these results are inconclusive, providing evidence instead for H_0_. In the absence of an objective effect, subjective information has the potential to be a better predictor of results, and this should be considered by investigators. To wit, even though this was not seen with our data, if a study is inadequately blinded then it is plausible that subjective experience *may* lead to significant differences in performance, and thus lead to spurious effects where no active control condition is included.

## Reanalysis 2: Filmer et al.^36^

### Methods

The method was identical to re-analysis 1 except where noted.

#### Data and materials

The study by Filmer et al.^36^ was pre-registered via the Open Science Framework with details of analysis plan, methodology and sample size by the authors (osf.io/zvqjb). The raw data from the original study has been made open source by the authors and can be accessed via the UQ eSpace^41^. For a full overview of the original methods, refer to Filmer et al.^36^.

#### Subjects

One hundred and fifty subjects were recruited for the study (mean age = 22.51, SD = 4.49 and 89 females). Three participants were removed from the initial data set due to equipment malfunctions but were replaced (N = 150). One participant was removed before the re-analysis (N = 149) for guessing both active and sham for subjective intervention.

#### Task^36^

The SART (Sustained Attention to Response Task) was used. Each block consisted of 6 trials, with each block containing three presentations of the target number and three presentations of the thought probe. Participants completed a short practice block with two trials: responding to one target number and one thought probe, before receiving tDCS. After stimulation, participants completed 8 blocks of the task, each separated by a short break.

#### tDCS

To denote electrode placement in reporting the stimulation polarities are marked as ‘+’ for anodal and ‘−’ for cathodal. The target regions are referred to as PFC (prefrontal cortex) and IPL (inferior parietal lobule). Two polarities were implemented: 1 mA and 2 mA. There were five experimental groups; (1) anode/cathode over the left PFC/right IPL at 1 mA (1 mA +PFC/−IPL), (2) anode/cathode over the left PFC/right IPL at 2 mA (2 mA +PFC/−IPL), (3) cathode/anode over the left PFC/right IPL at 1 mA (1 mA +IPL/−PFC), (4) cathode/anode over the left PFC/right IPL at 2 mA (2 mA +IPL/−PFC) and (5) sham stimulation with alternating electrode placement and dosages of the active stimulation groups.

#### Measures

##### Objective intervention

Refers to the condition in which the participant was assigned to one out of the five tDCS conditions.

##### Polarity

Refers to the polarity of the stimulation configuration, depending on where the cathode and anode are placed. Cathodal polarity consists of the 1 mA +IPL/−PFC and 2 mA +IPL/−PFC conditions, and anodal polarity consists of the 1 mA +PFC/−IPL and 2 mA +PFC/−IPL conditions.

##### Dosage

Refers to the strength of stimulation administered during the stimulation session: 1 mA and 2 mA.

#### Statistical analysis

The original findings of Filmer et al.^36^ were first replicated, before a series of one-way Bayesian ANOVAs were conducted to investigate the effect of both objective and subjective stimulation on TUT ratings regarding active conditions relative to sham, dosage, polarity and subjective stimulation intensity. Similar analyses were conducted to investigate the effect of stimulation on Target Accuracy, Non-Target Accuracy and Non-Target Reaction time. One-way ANOVAs were also conducted to investigate the best model fit when comparing objective stimulation, subjective stimulation, and an objective + subjective stimulation for TUT ratings. All relevant post-hoc pairwise comparisons were conducted following each analysis. The same categorisation of results for the Bayesian statistics will be used as in re-analysis 1, as outlined under ‘Statistical analysis’.

## Results

First and second half analyses were conducted to investigate the relationship between stimulation and mind-wandering over time, as it would be expected that the effects of stimulation would be strongest in the first half (first four blocks) as these are closest to the application of the stimulation, as compared to the second half (last four blocks). The results of these analyses followed the pattern of results as outlined below and have thus not been included for brevity. The results below feature the average of each dependent measure over all eight blocks.

### Effect of objective stimulation

The results of Filmer et al.^36^ were replicated. Relative to sham, a one-way Bayesian ANOVA with stimulation group as the between-subjects factor and average TUT-ratings as the dependent measure revealed anecdotal evidence for an overall main effect of objective group intervention (BF_10_ = 1.680, *F*(4,145) = 2.971, *p* = 0.021). Follow-up *t-tests* revealed that for stimulation effects relative to sham, only the 1 mA +PFC/−IPL (BF_10_ = 9.841, *t*(58) = 2.998, *p* = 0.004) and 2 mA +PFC/−IPL (BF_10_ = 2.721, *t*(58) = 2.389, *p* = 0.020) configurations demonstrated moderate and anecdotal evidence for stimulation effects respectively. The other two conditions 1 mA +IPL/−PFC (BF_10_ = 0.359, *t*(58) = 0.864, *p* = 0.391) and 2 mA +IPL/−PFC (BF_10_ = 0.447, *t*(58) = 1.127, *p* = 0.265) failed to reach evidential value (BF_10_ ~ 1 or above) in favour of H_1_. These results demonstrate an anecdotal to moderate effect of increased TUT-ratings after anodal stimulation to the left PFC and cathodal to the right IPL but no effects of cathodal stimulation to the left PFC with anodal stimulation to the right IPL.

However when investigating polarity specific effects, there was only anecdotal evidence for a polarity effect at 1 mA in 1 mA +PFC/−IPL and 1 mA +IPL/−PFC (BF_10_ = 1.657, *t*(58) = 2.113, *p* = 0.039) conditions. No evidence of polarity effects for the 2 mA (2 mA +PFC/−IPL and 2 mA +IPL/−PFC; BF_10_ = 0.571, *t*(58) = 1.364, *p* = 0.178) were found. Similarly, no effect of dosage was found for either configuration (+IPL/−PFC; BF_10_ = 0.268, *t*(58) = − 0.232, *p* = 0.817, +PFC/−IPL; BF_10_ = 0.290, *t*(58) = 0.489, *p* = 0.627).

### Comparing the effect of objective and subjective intervention

One-way Bayesian ANOVAs evaluated the effect of Objective Intervention, Subjective Intervention, and a combined measure of Objective and Subjective Intervention on average TUT-ratings. This was done individually for each of the four stimulation conditions as compared to sham and the full model for each comparison can be found in Appendix C and D. The strongest evidence was seen for 1 mA +PFC/−IPL configuration as compared to sham for objective intervention (BF_inc_ = 8.811, BF_10_ = 9.840, *F*(1, 56) = 7.117, *p* = 0.010), demonstrating moderate evidence for accounting for the variance seen over and above that of subjective intervention alone (BF_inc_ = 0.296, BF_10_ = 0.431, *F*(1, 56) = 0.002, *p* = 0.964), or the combined variable (see Appendix C1). For the 2 mA +PFC/−IPL configuration, there was anecdotal evidence for objective intervention (BF_inc_ = 2.659, BF_10_ = 2.463, *F*(1, 55) = 8.032, *p* = 0.006) accounting for the variance as compared to sham over and above that of subjective intervention (BF_inc_ = 0.343, BF_10_ = 0.271, *F* (1, 55) = 1.525, *p* = 0.222) or the combined variable of objective and subjective intervention (see Appendix C2). This follows the pattern of results from the initial findings of Filmer et al.^36^, where only the configurations with anodal stimulation to the left PFC demonstrated meaningful results. It also mirrors the results of re-analysis 1, in which when comparing the effects of a specific stimulation group that demonstrated an objective effect of stimulation with subjective intervention, the variance is predominately attributed to the objective intervention.

No evidence of effects were seen for the sham versus 2 mA +IPL/−PFC configuration for objective (*F*(1, 56) = 2.210, *p* = 0.143) or subjective (*F*(1, 56) = 0.733, *p* = 0.396) intervention nor were there effects for the combined variable (see Appendix C3). Similarly for the 1 mA +IPL/−PFC configuration, there were no effects of objective (*F*(1, 56) = 0.581, *p* = 0.449), subjective stimulation (*F*(1, 56) = 0.059, *p* = 0.809) or the combined variable when compared in a model (Appendix C4). This is unsurprising, given there were no effects of stimulation seen for either configuration in the initial analysis.

For the interest of consistency polarity and dosage were also evaluated even though no strong evidence was found in the initial analyses of the effects of stimulation. Regarding dosage, objective (*F*(1, 55) = 0.008, *p* = 0.927), subjective (*F*(1, 55) = 1.520, *p* = 0.223) and the combined variable (*F*(1, 55) = 0.978, *p* = 0.327) provided BF_10_ values that either did not surpass the criteria of inconclusive (BF_10_ 1–3) or provided evidence for H_0_ over H_1_ for the +PFC/−IPL configurations (1 mA and 2 mA), see Appendix D1 for the full model. Similarly for the +IPL/−PFC configurations (1 mA and 2 mA), all three predictors failed to reach a BF value greater than BF_10_ ~ 1 (subjective; *F*(1, 56) = 0.626 *p* = 0.432), objective; *F*(1, 56) = 0.459 *p* = 0.501, or combined; *F*(1, 56) = 1.068, *p* = 0.306), as seen in Appendix D2.

Lastly, polarity showed anecdotal evidence for configurations at 1 mA (1 mA +PFC/−IPL and 1 mA +IPL/−PFC), as anecdotal evidence was found for an effect of objective intervention (BF_inc_ = 1.641, BF_10_ = 1.657, *F* (1, 56) = 3.644, *p* = 0.061) over and above that of subjective intervention (BF_inc_ = 0.294, BF_10_ = 0.302, *F* (1, 56) = 0.001, *p* = 0.975) or combined objective and subjective intervention (Appendix D3). For the configurations at 2 mA (2 mA +PFC/−IPL and 2 mA +IPL/−PFC), anecdotal evidence was demonstrated for subjective intervention (BF_inc_ = 1.533, BF_10_ = 1.471, *F* (1, 55) = 4.298, *p* = 0.043) over and above that of objective intervention alone (BF_inc_ = 0.590, BF_10_ = 0.551, *F* (1, 55) = 2.130, *p* = 0.150) or combined objective and subjective intervention (Appendix D4). However, no conclusions can be drawn from these findings, given the relatively low strength of the BF_inc_ values.

### Effect of subjective intervention

When evaluating the effect of subjective intervention, one-way Bayesian ANOVAs were used. Across all investigated measures (relative to sham, dosage, polarity, and subjective stimulation intensity) there was little to no evidence of an effect of subjective intervention on average TUT-ratings. Participant ratings of whether they were in the active or sham condition had no effect on average TUT-rating (BF_10_ = 0.196, *F*(1, 147) = 0.102, *p* = 0.750). When evaluating specific dosage conditions, no evidence of an effect of subjective belief of active or sham condition was found for both the +PFC/−IPL (BF_10_ = 0.579, *t*(57) = 1.266, *p* = 0.211) or the +IPL/—PFC configurations (BF_10_ = 0.347, *t*(58) = 0.713, *p* = 0.479). Regarding polarity, at best only anecdotal evidence was observed for both polarities (1 mA +PFC/−IPL vs. 1 mA +IPL/−PFC 1 mA; BF_10_ = 0.302, *t*(58) = − 0.315, *p* = 0.754) (2 mA +PFC/−IPL vs. 2 mA +IPL/−PFC; BF_10_ = 1.471, *t*(58) = 2.009, *p* = 0.049) and thus no conclusions can be drawn about the effect of subjective intervention for polarity.

The effect of subjective stimulation intensity on average TUT-rating was also evaluated however no effect was found (BF_10_ = 0.061, *F*(3, 145) = 0.084, *p* = 0.969), regardless of a participants subjective belief of the strength of stimulation they received (very weak, weak, moderate or strong).

Similar analyses were conducted to investigate the effect of stimulation on Target Accuracy (BF_10_ = 0.348, *F*(3, 145) = 1.366, *p* = 0.244), Non-Target Accuracy (BF_10_ = 0.190, *F*(3, 145) = 0.031, *p* = 0.861) and Non-Target Reaction time (BF_10_ = 0.286, *F*(3, 145) = 0.932, *p* = 0.336) however, again, no significant effects were found.

## Discussion

Blinding efficacy has been called into question and reporting of correct guess rate, a common practice in NIBS research, has been highlighted to have possible shortcomings^31^ in its ability to provide insight into the success (or failure) of blinding. There have been examples of methods^31,34^ that could provide better insight into this issue, one such method being that of Fassi and Kadosh^35^, which was employed to examine the effect of subjective belief, particularly the hypothesis that participants’ subjective beliefs about the influence of tDCS on a cognitive task drove the previous results of Filmer et al.^4^ on mind-wandering. The authors concluded that subjective intervention, a participant’s personal belief about which stimulation condition they received, was a significant predictor of mind-wandering over and above that of objective intervention. Similarly, when investigating dosage, it was concluded that subjective dosage, a participant’s personal belief regarding the intensity of stimulation they received was also a better predictor of mind-wandering than objective intervention alone.

However, it can be argued that this was misleading given that Fassi and Kadosh^35^ looked at all conditions from Filmer et al.^4^ together when only one stimulation condition, cathodal 2.0 mA, produced meaningful effects regarding mind-wandering. In short, it was problematic to evaluate the effect of subjective belief across all stimulation conditions given that this fails to follow up the previous key result. The present study sought to investigate whether the effects described regarding subjective stimulation held once individual key effects were investigated in a re-analysis. In addition, we investigated the effect subjective intervention could have in the re-analysis of another mind-wandering data set from our lab featuring the same SART methodology.

When evaluating *only* cathodal 2.0 mA compared to sham from Filmer et al.^4^ the effects of subjective beliefs about both intervention and dosage no longer are found. Specifically, we observed moderate evidence to support the notion that objective intervention is the strongest predictor of mind-wandering with higher rates of mind-wandering observed within the cathodal 2.0 mA condition relative to sham. Interestingly, subjective intervention became the weakest predictor within the model, lesser than that of objective intervention, objective intervention and subjective intervention combined or an interaction of the two.

The same pattern of results was seen for the second re-analysis. Specifically, the key condition that had originally been reported as showing an effect on mind wandering (1 mA +PFC/−IPL) provided moderate evidence for objective intervention as the strongest predictor over and above that of subjective intervention alone or a combination of the two. All other configurations, and the investigation of polarity and dosage failed to demonstrate a meaningful effect of subjective intervention. When you consider the findings from both studies, it is clear that for at least these two mind-wandering studies, participant subjective belief regarding the intervention they received had no effect on the observed changes in mind-wandering.

Despite the present results, the concerns raised about subjective participant beliefs are warranted. Given the thought-provoking critique of the current standard methods of blinding within the literature, more sensitive measures and tests are needed to investigate issues of blinding and subjective belief. Indeed, further investigation of previous data sets and studies within the literature is needed to determine whether these findings have been influenced by participant belief.

Future studies should implement measures that aim to reduce the effects of subject belief and improve the blinding methods. There is the possibility of implementing methods such as anaesthetic creams to remove the cutaneous feelings entirely from both active and sham conditions, a method that has been successfully used in both animal studies using transcranial alternating current stimulation (tACS)^28^—a similar method to tDCS that involves an oscillating electrical current rather than a direct current^43^—and in humans during tDCS^22,29^. Another consideration is participant experience level, given the findings of Ambrus et al.^21^, who observed that participants who had previous experience with tDCS were more likely to correctly identify trials which featured stimulation and those that featured sham whereas naïve participants were less likely to correctly identify their stimulation condition. This is relatively easy to control through screening processes during recruitment that could help to reduce the unblinding of participants.

Relying on the comparison of a single target condition to a sham condition can be problematic if effective blinding is not achieved, particularly at the participant level as typically participants guess above chance in identifying stimulation conditions^4^. Further, the points raised by Fassi and Kadosh^35^ and Filmer et al.^4^, as discussed earlier, support the need for the departure from solely sham controlled methods. The use of active controls or a combination of both active and sham-controlled methods would avoid the issue of unblinding due to perceptual sensation. Active controls also allow for the investigation of the specific role of targeted brain regions when a separate brain region is targeted in the control, or polarity effects when an alternative polarity is applied to the control region^3^. Given the possibility to also investigate hemispheric differences, active controls can provide both a rich comparative measure and interesting investigative opportunities. However, when comparing two active conditions it can be difficult to distinguish which condition is modulating the behaviour when there is a difference between the two conditions, or whether both have an effect. Thus, the most beneficial configuration would be to include a condition of interest, a sham condition and at least one other active control condition, to help mitigate the pros and cons of each technique.

We acknowledge some limitations. First, Filmer et al.^4^ did not feature an active control and thus (as outlined previously) is subject to inefficient blinding. We have previously addressed these issues in an opinion paper discussing tDCS efficacy as whole^3^ , as well as in subsequent papers. Although no a priori active control conditions were included for either Filmer et al.^4^ or Filmer et al.^36^ some conclusions can be made about the other active protocols. The ‘ineffective’ active protocols for Filmer et al.^4^ such as the anodal 1.0 mA, cathodal 1.0 mA and cathodal 1.5 mA which demonstrated statistically negligible outcomes on mind-wandering can also be compared to key active conditions. Similarly for the second re-analysis, the findings for +IPL/−PFC were non-substantial and thus can also be compared in this way. Here there is potential for these protocols (and ineffective active protocols in general) to be regarded as active controls, given the statistical differences with key active conditions. Specifically, if there were to be an effect of subjective participant belief on stimulation effects on mind-wandering due to a failure of blinding, it would be expected that these beliefs would generalise to all active conditions, not just one key condition.

However, another limitation to note with this kind of comparison, and of our re-analyses, is the lack of active conditions that differ from one another. The findings of the present re-analysis demonstrate one active group (cathodal 2.0 mA and +PFC/−IPL) that differs from sham, but not an active group that differs from another active group, thus making it difficult to draw definitive conclusions about the role of ‘ineffective active protocol’ controls.

It is also recognised that the analyses of Fassi and Kadosh^35^ were based on the omnibus findings of Filmer et al.^4^ and could be argued to follow the pre-registered analysis plan of our original paper more closely. To mediate this issue for the present study, we added a second data set which implements the same stimulation dosages (intensities and duration) as well as the same mind-wandering measures as Filmer et al.^4^. Nowhere do we claim that our blinding method is without fault however after controlling for both objective and subjective intervention, it appears that at least within these two data sets, participant subjective belief of intervention does not meaningfully influence the effects of stimulation on mind-wandering.

The insights and discussions outlined in the present study are facilitated by open science practices and underscore the importance and utility of sharing data. Without these practices, the field would continue to stagnate with poor methodological methods, particularly ineffective blinding, and the discussion of future improvements would be limited. Here we have made the results of Filmer et al.^4^ and Filmer et al.^36^ more definitive, however this would not have been possible with the insights of Fassi and Kadosh^35^ highlighting the benefits of multilab collaboration.

## Supplementary Information

Supplementary Information.

## Data Availability

The dataset analysed in re-analysis one is available via the UQ eSpace repository [10.14264/uql.2019.295]. The dataset analysed in re-analysis two is available via the UQ eSpace repository [10.48610/2e9f4ef].
