# Supplementary material for: Intervention is a better predictor of tDCS mind-wandering effects than subjective beliefs about experimental results
Source: Sci Rep. 2022 Jul 30;12:13110. doi: 10.1038/s41598-022-16545-0 (PMC9338927; doi:10.1038/s41598-022-16545-0)
Supplement: Supplementary file 1 — Supplementary Information. [file 41598_2022_16545_MOESM1_ESM.docx]

**Appendix A**

| **Models** | **P(M)** | **P(M \| Data)** | **BF_10_** | **Error %** |
| --- | --- | --- | --- | --- |
| *Subjective Intervention* | 0.200 | 0.464 | 3.374 | 8.94 e^-8^ |
| *Objective +Subjective Intervention* | 0.200 | 0.205 | 1.492 | 2.201 |
| *Null Model* | 0.200 | 0.137 | 1.000 |  |
| *Objective* | 0.200 | 0.135 | 0.984 | 1.40 e^-4^ |
| *Objective * Subjective Intervention* | 0.200 | 0.059 | 0.429 | 1.000 |

Appendix A1. Bayesian ANOVA comparing Objective and Subjective Intervention for best model fit using average TUT score as the outcome measure ^[35]^ .

| **Models** | **P(M)** | **P(M \| Data)** | **BF_10_** | **Error %** |
| --- | --- | --- | --- | --- |
| *Subjective Dosage* | 0.200 | 0.489 | 3.708 | 1.715 e^-4^ |
| *Objective Intervention +Subjective Dosage* | 0.200 | 0.219 | 1.658 | 0.587 |
| *Null Model* | 0.200 | 0.132 | 1.000 |  |
| *Objective* | 0.200 | 0.130 | 0.984 | 1.402 e^-4^ |
| *Objective Intervention * Subjective Dosage* | 0.200 | 0.031 | 0.232 | 0.582 |

Appendix A2. Bayesian ANOVA comparing Objective and Subjective Dosage for best model fit using average TUT score as the outcome measure ^[35]^.

**Appendix B**

| **Models** | **P(M)** | **P(M \| Data)** | **BF_10_** | **BF_inc_** | **Error %** |
| --- | --- | --- | --- | --- | --- |
| *Null Model* | 0.200 | 0.376 | 1.000 |  |  |
| *Objective Intervention* | 0.200 | 0.639 | 7.436 | 7.437 | 8.694 e^-5^ |
| *Objective Intervention + Subjective Intervention* | 0.200 | 0.183 | 2.133 | 0.365 | 2.627 |
| *Objective + Subjective Intervention + Objective Intervention * Subjective Intervention* | 0.200 | 0.069 | 0.798 | - | 4.306 |
| *Subjective Intervention* | 0.200 | 0.024 | 0.276 | 0.276 | 0.004 |

Appendix B1. Bayesian ANOVA comparing Objective and Subjective Intervention with the stimulation conditions Cathodal 2.0mA and Sham for best model fit using average TUT score as the outcome measure.

| **Models** | **P(M)** | **P(M \| Data)** | **BF_10_** | **BF_inc_** | **Error %** |
| --- | --- | --- | --- | --- | --- |
| *Null Model* | 0.200 | 0.376 | 1.000 |  |  |
| *Objective Intervention* | 0.200 | 0.709 | 7.436 | 6.874 | 8.694 e^-5^ |
| *Objective Intervention + Subjective Dosage* | 0.200 | 0.115 | 1.203 | 0.510 | 2.011 |
| *Objective Intervention + Subjective Dosage + Objective Intervention * Subjective Dosage* | 0.200 | 0.057 | 0.599 | - | 1.246 |
| *Subjective Dosage* | 0.200 | 0.024 | 0.254 | 0.170 | 0.033 |

Appendix B2. Bayesian ANOVA comparing Objective Intervention and Subjective Dosage with the stimulation conditions Cathodal 2.0mA and Sham for best model fit using average TUT score as the outcome measure.

**Appendix C**

| **Models** | **P(M)** | **P(M \| Data)** | **BF_incl_** | **BF_10_** | **Error %** |
| --- | --- | --- | --- | --- | --- |
| *Null Model* | 0.200 | 0.066 |  | 1.000 |  |
| *Objective Intervention* | 0.200 | 0.652 | 8.811 | 9.840 | 2.285e^-5^ |
| *Objective +Subjective Intervention* | 0.200 | 0. 184 |  | 2.772 | 1.609 |
| *Subjective Intervention* | 0.200 | 0.029 | 0.296 | 0.431 | 0.010 |
| *Objective * Subjective Intervention* | 0.200 | 0.070 | 0.382 | 1.059 | 1.126 |

Appendix C1. Bayesian ANOVA comparing Objective and Subjective Intervention for best model fit using average TUT score as the outcome measure in the key contrast 1mA +PFC/ -IPL vs. sham.

| **Models** | **P(M)** | **P(M \| Data)** | **BF_incl_** | **BF_10_** | **Error %** |
| --- | --- | --- | --- | --- | --- |
| *Null Model* | 0.200 | 0.188 |  | 1.000 |  |
| *Objective Intervention* | 0.200 | 0.463 | 2.659 | 2.463 | 0.005 |
| *Objective +Subjective Intervention* | 0.200 | 0. 172 |  | 0.917 | 1.315 |
| *Subjective Intervention* | 0.200 | 0.051 | 0.343 | 0.271 | 0.007 |
| *Objective * Subjective Intervention* | 0.200 | 0.126 | 0.732 | 0.671 | 0.974 |

Appendix C2. Bayesian ANOVA comparing Objective and Subjective Intervention for best model fit using average TUT score as the outcome measure in the key contrast 2mA +PFC/ -IPL vs. sham

| **Models** | **P(M)** | **P(M \| Data)** | **BF_incl_** | **BF_10_** | **Error %** |
| --- | --- | --- | --- | --- | --- |
| *Null Model* | 0.200 | 0.510 |  | 1.000 |  |
| *Objective Intervention* | 0.200 | 0.228 | 0.462 | 0.447 | 0.005 |
| *Objective +Subjective Intervention* | 0.200 | 0.074 |  | 0.145 | 0.944 |
| *Subjective Intervention* | 0.200 | 0.143 | 0.294 | 0.280 | 0.005 |
| *Objective * Subjective Intervention* | 0.200 | 0.045 | 0.610 | 0.089 | 14.480 |

Appendix C3. Bayesian ANOVA comparing Objective and Subjective Intervention for best model fit using average TUT score as the outcome measure in the key contrast 2mA +IPL/ -PFC vs. sham.

| **Models** | **P(M)** | **P(M \| Data)** | **BF_incl_** | **BF_10_** | **Error %** |
| --- | --- | --- | --- | --- | --- |
| *Null Model* | 0.200 | 0.562 |  | 1.000 |  |
| *Objective Intervention* | 0.200 | 0.202 | 0.357 | 0.359 | 0.003 |
| *Objective +Subjective Intervention* | 0.200 | 0.056 |  | 0.100 | 2.885 |
| *Subjective Intervention* | 0.200 | 0.161 | 0.284 | 0.286 | 0.003 |
| *Objective * Subjective Intervention* | 0.200 | 0.019 | 0.336 | 0.034 | 1.978 |

Appendix C4. Bayesian ANOVA comparing Objective and Subjective Intervention for best model fit using average TUT score as the outcome measure in the key contrast 1mA +IPL/ -PFC vs. sham.

**Appendix D**

| **Models** | **P(M)** | **P(M \| Data)** | **BF_incl_** | **BF_10_** | **Error %** |
| --- | --- | --- | --- | --- | --- |
| *Null Model* | 0.200 | 0.467 |  | 1.000 |  |
| *Objective Intervention* | 0.200 | 0.137 | 0.294 | 0.293 | 0.004 |
| *Objective +Subjective Intervention* | 0.200 | 0.080 |  | 0.172 | 0.920 |
| *Subjective Intervention* | 0.200 | 0.270 | 0.580 | 0.579 | 0.010 |
| *Objective * Subjective Intervention* | 0.200 | 0.046 | 0.567 | 0.097 | 2.329 |

Appendix D1. Bayesian ANOVA comparing Objective and Subjective Intervention for best model fit using average TUT score as the outcome measure to investigate dosage with the +PFC/-IPL configurations (1mA and 2mA).

| **Models** | **P(M)** | **P(M \| Data)** | **BF_incl_** | **BF_10_** | **Error %** |
| --- | --- | --- | --- | --- | --- |
| *Null Model* | 0.200 | 0.570 |  | 1.000 |  |
| *Objective Intervention* | 0.200 | 0.153 | 0.267 | 0.268 | 0.003 |
| *Objective +Subjective Intervention* | 0.200 | 0.052 |  | 0.092 | 2.981 |
| *Subjective Intervention* | 0.200 | 0.198 | 0.346 | 0.347 | 0.019 |
| *Objective * Subjective Intervention* | 0.200 | 0.027 | 0.521 | 0.048 | 3.624 |

Appendix D2. Bayesian ANOVA comparing Objective and Subjective Intervention for best model fit using average TUT score as the outcome measure to investigate dosage with the +IPL/-PFC configurations (1mA and 2mA).

| **Models** | **P(M)** | **P(M \| Data)** | **BF_incl_** | **BF_10_** | **Error %** |
| --- | --- | --- | --- | --- | --- |
| *Null Model* | 0.200 | 0.276 |  | 1.000 |  |
| *Objective Intervention* | 0.200 | 0.458 | 1.641 | 1.657 | 0.007 |
| *Objective +Subjective Intervention* | 0.200 | 0.132 |  | 0.479 | 0.877 |
| *Subjective Intervention* | 0.200 | 0.083 | 0.294 | 0.302 | 0.022 |
| *Objective * Subjective Intervention* | 0.200 | 0.051 | 0.392 | 0.183 | 1.165 |

Appendix D3. Bayesian ANOVA comparing Objective and Subjective Intervention for best model fit using average TUT score as the outcome measure to investigate polarity via the 1mA configurations (+PFC/-IPL 1mA and +IPL/-PFC 1mA**)**

| **Models** | **P(M)** | **P(M \| Data)** | **BF_incl_** | **BF_10_** | **Error %** |
| --- | --- | --- | --- | --- | --- |
| *Null Model* | 0.200 | 0.234 |  | 1.000 |  |
| *Objective Intervention* | 0.200 | 0.129 | 0.590 | 0.551 | 0.008 |
| *Objective +Subjective Intervention* | 0.200 | 0.212 |  | 0.907 | 0.757 |
| *Subjective Intervention* | 0.200 | 0.344 | 1.533 | 1.471 | 6.900 e^-4^ |
| *Objective * Subjective Intervention* | 0.200 | 0.081 | 0.383 | 0.347 | 1.060 |

Appendix D4. Bayesian ANOVA comparing Objective and Subjective Intervention for best model fit using average TUT score as the outcome measure to investigate polarity via the 2mA configurations (+PFC/-IPL 2mA and +IPL/-PFC 2mA**)**
